# Genetic variation and recombination analysis of the *GP5* gene of the porcine reproductive and respiratory syndrome virus in Thailand

**DOI:** 10.3389/fvets.2024.1444040

**Published:** 2024-08-08

**Authors:** Yajie Zheng, Gan Li, Kexin Liu, Qin Luo, Wenchao Sun, Mengmeng Zhao

**Affiliations:** ^1^Guangdong Provincial Key Laboratory of Animal Molecular Design and Precise Breeding, School of Life Science and Engineering, Foshan University, Foshan, China; ^2^Wenzhou Key Laboratory for Virology and Immunology, Institute of Virology, Wenzhou University, Wenzhou, China

**Keywords:** porcine reproductive and respiratory syndrome virus in Thailand, *GP5* gene, genetic variation, phylogeny, recombination

## Abstract

**Introduction:**

Porcine reproductive and respiratory syndrome (PRRS) is a significant threat to the global swine industry, and its prevalence in Thailand spans over two decades.

**Methods:**

To understand the genetic variation and recombination of the PRRS virus (PRRSV) *GP5* gene in Thailand, we retrieved 726 *GP5* gene sequences from the NCBI database. Phylogenetic trees were constructed using the neighbor-joining (NJ) and maximum likelihood (ML) methods, and recombination analysis was performed.

**Results:**

Homology analysis was conducted on 83 PRRSV-1 and 83 PRRSV-2 strains. Phylogenetic analysis revealed the prevalence of both PRRSV-1 and PRRSV-2 strains in Thailand, with the latter exhibiting wider distribution. PRRSV-1 strains clustered into clades A, D, and H, while PRRSV-2 strains grouped into lineages 1, 5, and sublineage 8.7, further divided into 8.7/HP and 8.7/NA sublineages. Sublineage 8.7/NA strains accounted for a significant proportion of circulating PRRSV-2 strains. Homology analysis showed nucleotide and amino acid similarities ranging from 75.4 to 100.0% and 41.3 to 100.0% for PRRSV-1, and 78.6 to 100.0% and 70.8 to 100.0% for PRRSV-2 strains. Amino acid sequence alignments revealed mutations, insertions, and deletions in PRRSV-1 GP5, and key residue mutations in PRRSV-2 GP5 associated with biological functions. Recombination analysis identified two recombination events within PRRSV-2 sublineage 8.7 strains.

**Discussion:**

These findings confirm the variability of the GP5 protein. This study enhances our understanding of PRRSV prevalence and genetic variation in Thailand, contributing valuable insights for PRRS prevention and control.

## 1 Introduction

Porcine reproductive and respiratory syndrome (PRRS), a highly contagious disease caused by the PRRS virus (PRRSV), can lead to reproductive disorders in sows, severe respiratory diseases, and impaired growth performance in piglets and growing pigs, thereby resulting in significant economic losses to the global pig industry (1). PRRS was initially discovered in central Europe and North America during the late 1980s, and has subsequently spread worldwide (2–4). In the US alone, this disease has caused economic loss of $664 million to the pig industry every year, with losses in excess of €1.5 billion in Europe, and significant damage in China (5–7). Furthermore, a study conducted in the Netherlands found that the cost per sow due to different approaches in managing PRRS outbreaks ranged from €3 to €160 (8). The earliest detection of PRRSV seropositive animals in Thailand can be traced back to 1989, and the first isolation of a genotype NA strain of PRRSV in Thailand was reported in 1996 (9). Subsequently, the rate of PRRSV seropositivity in Thailand increased annually between 1991 and 2002 (9), whereas in 2004, Type 1 PRRSV strains were also reported in Thailand, with co-circulation of PRRSV-1 and PRRSV-2 strains within the same pig herds being detected (10). In 2006, an outbreak of highly pathogenic PRRSV (HP-PRRSV) caused substantial damage to the Chinese pig industry, affecting a majority of the domestic pig herds and spreading to neighboring countries (11, 12), among which the Laos People’s Democratic Republic was infected with HP-PRRSV in 2008, followed 2 years later by Thailand (13, 14).

PRRSV is an enveloped, positive-sense, single-stranded RNA virus in the family *Arteriviridae* of the order *Nidovirales*. It has a genome of approximately 15 kb in length, which encodes at least 22 different viral proteins, of which 14 and 8 are non-structural and structural proteins, respectively, with the latter being designated ORF2a/ORF2b, ORF3, ORF4, ORF5/ORF5a, ORF6, and ORF7. Among these, ORF5/ORF5a respectively encode the GP5 and GP5a proteins (15–19). PRRSV is broadly classified into two genotypes, namely, PRRSV-1 (European type) and PRRSV-2 (North American type), which are also respectively referred to as *Betaarterivirus suid 1* and *Betaarterivirus suid 2*, that have a genetic similarity as high as 60% (20–22). Currently, both genotypes are circulating in pig populations worldwide, and have been established to be rapidly evolving and display high genetic variability (23–25). Moreover, pathogenic strains have emerged among both types, thereby posing a constant threat to the global pig industry (11, 26, 27).

GP5 is the most abundant glycoprotein found on the surface of viral particles, and is hence referred to as the major envelope glycoprotein. The sequence of GP5 contains approximately 200 amino acids comprising a transmembrane region and an N-terminal extracellular domain, containing multiple antibody-neutralizing epitopes (28, 29). On the basis of amino acid sequence predictions, the GP5 protein is predicted to contain three or four transmembrane domains, along with a cleavable signal peptide at the N terminus that guides protein synthesis to the rough endoplasmic reticulum (30). Furthermore, its N-glycosylation sites have been established to be associated with viral neutralization and pathogenicity (31). The extracellular domain of GP5 consists of 35 amino acids, including two to five potential N-linked glycosylation sites, which constitute antibody-neutralizing epitopes that are common features among PRRSV isolates (32). Moreover, the GP5 protein has been identified one of the most variable proteins in PRRSV, and this high variability is often used as a basis for phylogenetic analysis and studies of genetic evolution (33).

In this study, we used 678 PRRSV *GP5* gene sequences collected since the introduction of PRRSV into Thailand, along with 48 reference strains, to perform phylogenetic and recombination analyses, among which, we selected representative strains for subsequent nucleotide and amino acid sequence analyses to assess homologies and perform sequence alignments. On the basis of the findings of these analyses, we characterized the genetic variation and recombination events of PRRSV GP5 in Thailand, the information on which will contribute to facilitating the effective monitoring of PRRSV epidemiology in Thailand and the development of measures for the prevention and control of PRRS.

## 2 Materials and methods

### 2.1 Dataset

For the purposes of this study, we obtained a total of 726 PRRSV GP5 sequences from the NCBI Nucleotide database, including 194 sequences of Thai PRRSV-1 GP5, 484 sequences of Thai PRRSV-2 GP5, and 48 sequences from both PRRSV-1 and PRRSV-2 classical reference strains from other countries. Detailed information pertaining to these strains can be found in Supplementary Table S1. The selected strains corresponded to all Thai PRRSV GP5 sequences deposited in the NCBI Nucleotide database since the introduction of PRRSV into Thailand, along other country-specific vaccine strains and classical PRRSV strains as reference strains for epidemiological analysis. Among these 726 sequences, those of 83 PRRSV-1 strains and 83 PRRSV-2 strains were selected based on the following criteria: the frequency of being reported as representative PRRSV strains, the coverage of strains from different years, uniform selection within each lineage based on lineage classification to include representative strains from each lineage, and inclusion of 48 strains from other countries representing classical prevalent strains. Detailed information regarding the 166 selected strains is presented in Supplementary Table S2.

### 2.2 Phylogenetic analysis

Using the retrieved set of 726 PRRSV *GP5* gene sequences, we constructed phylogenetic trees. Initially, the Clustal W alignment tool in MEGA software (version 7.0.26; Mega Limited, Auckland, New Zealand) was employed to align the nucleotide sequences of the target gene, and thereafter, the neighbor-joining (NJ) and maximum likelihood (ML) methods with 1,000 and 100 bootstrap replicates, respectively, and default parameters, were used to construct phylogenetic trees. The resulting trees were exported to The Interactive Tree Of Life online software^1^ and subsequently annotated.

### 2.3 Analysis of GP5 nucleotide homology

To determine the nucleotide homology among the GP5 sequences of different PRRSV subtypes and lineages, we selected 83 sequences each of PRRSV-1 and PRRSV-2 GP5, and used the Clustal W method in MegAlign, a feature of DNASTAR software (version 7.0; Madison, WI), for analysis.

### 2.4 Analysis and GP5 amino acid homology and sequence alignment

To determine the amino acid homology among the selected 83 PRRSV-1 and PRRSV-2 GP5 sequences, we initially used the Editseq function in DNASTAR software (version 7.0; Madison, WI) to convert the nucleotide sequences to amino acid sequences, and same procedures as described for the nucleotide homology analysis were applied to analyze amino acid sequence homologies. Alignment of the PRRSV-1 and PRRSV-2 GP5 amino acid sequences was performed using the aforementioned Clustal W method.

### 2.5 Recombination analysis of GP5

Potential recombination events in GP5 were examined using seven different methods available in recombination detection program (RDP) software (version 4.0), namely, RDP, MaxChi, GeneConv, BootScan, SiScan, 3Seq, and Chimaera. Identification of potential recombination events was based on detection using three or more of these algorithms with a significance level of *p* < 0.05. In addition, the detected recombination events were further validated using SimPlot software (version 3.5.1).

## 3 Results

### 3.1 Phylogenetic analysis

**Figure 1:**
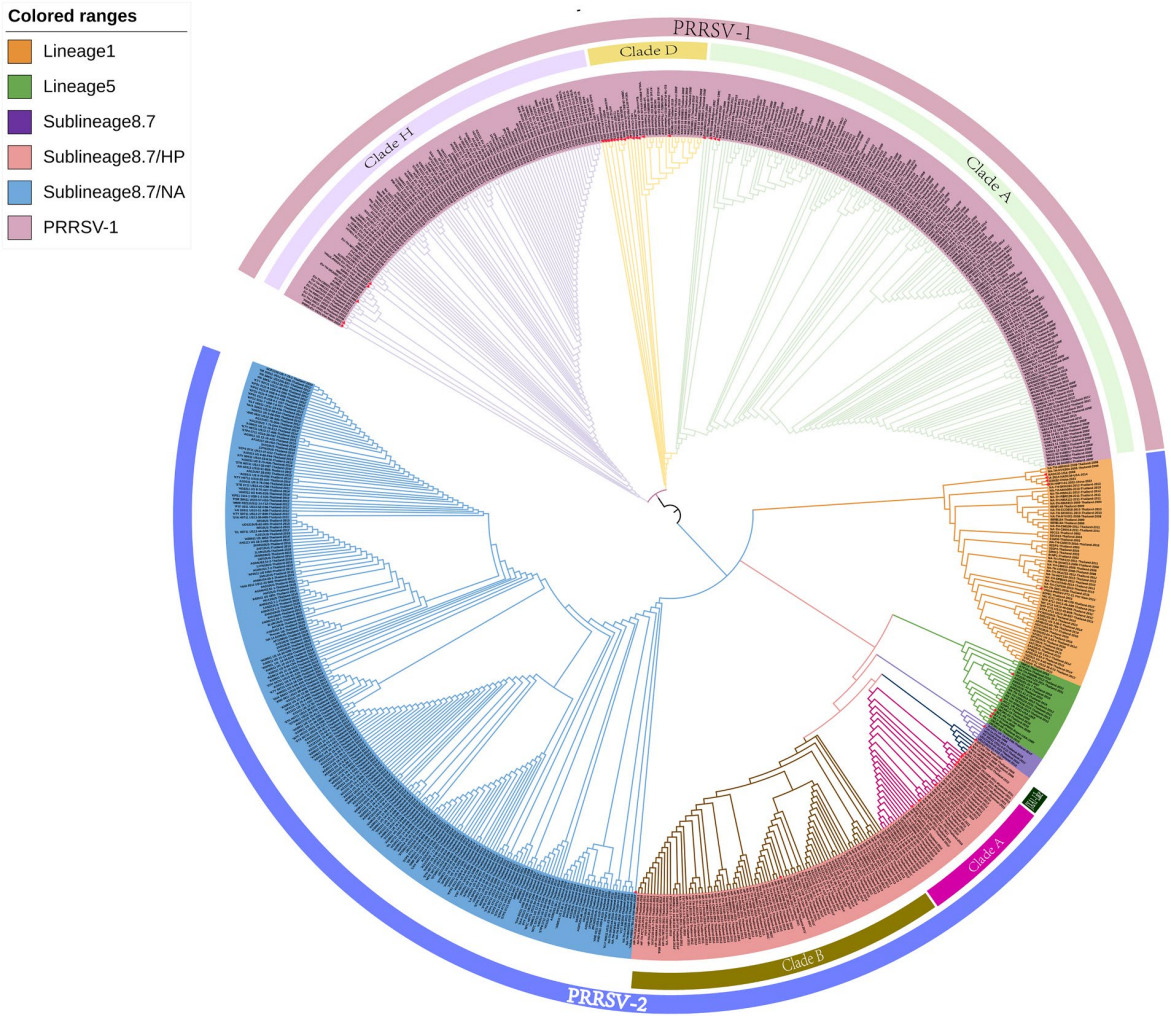
A neighbor-joining phylogenetic tree was constructed using MEGA software with 1,000 bootstrap replicates and default parameters based on 726 PRRSV GP5 sequences. The tree was annotated using The Interactive Tree of Life online software. Red triangles represent reference strains.

On the basis of the global PRRSV classification system and GP5 sequence information obtained from the GenBank database, we selected 726 PRRSV *GP5* gene sequences for the purposes of phylogenetic tree construction (Supplementary Table S1). The evolutionary tree constructed using the NJ method revealed that both PRRSV-1 and PRRSV-2 strains are prevalent in Thailand, with the latter strains being characterized by a wider prevalence (Figure 1). Among the PRRSV-1 strains circulating in Thailand, three evolutionary branches were identified, namely, clades A, D, and H, whereas the PRRSV-2 strains can be broadly divided into three lineages (lineages 1, 5, and sublineage 8.7). Among these lineages, sublineage 8.7 comprises sublineages 8.7/HP and 8.7/NA strains, the former of which can be sub-divided into clade A, clade B, and JXA1-like stains. Among PRRSV-2 strains in Thailand, those in sublineage 8.7/NA appear to be the most prevalent, and lineage 5 strains were established to be genetically close to those in sublineages 8.7 and 8.7/HP. Furthermore, we found that the PRRSV strains isolated in Thailand are genetically relatively close to selected reference strains isolated over the past 5 years.

**Figure 2:**
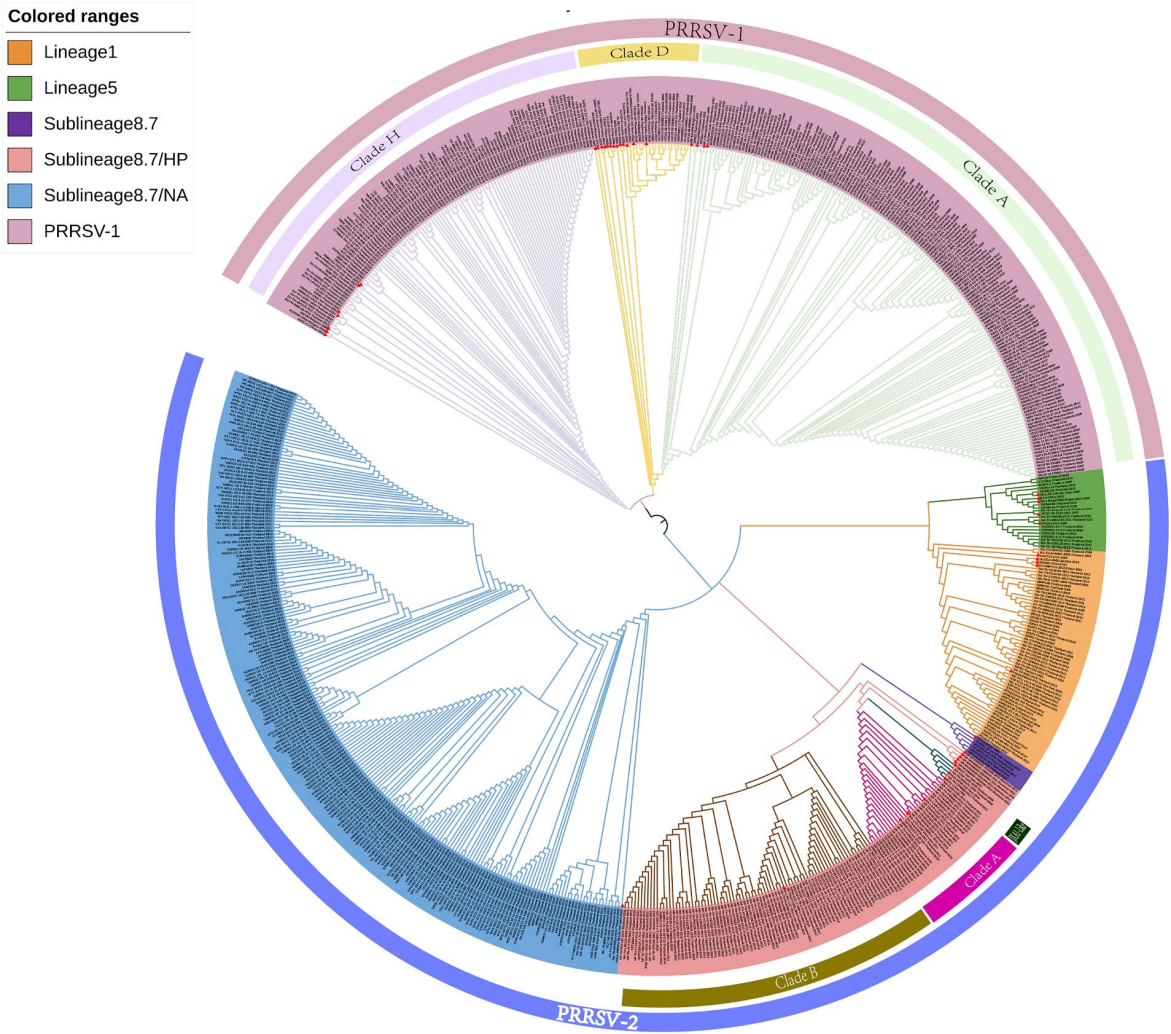
A maximum likelihood phylogenetic tree was constructed using MEGA software with 100 bootstrap replicates and default parameters based on 726 PRRSV GP5 sequences. The tree was annotated using The Interactive Tree of Life online software. Red triangles represent reference strains.

The clustering of strains in the evolutionary tree constructed using the ML method was found to be generally consistent with the results obtained using the NJ method, although there were also certain differences (Figure 2). For example, the ML tree revealed a greater genetic distance between reference strains from Myanmar and PRRSV-2 clade A and JXA1-like strains. In addition, whereas in the NJ phylogenetic tree, lineage 5 and sublineage 8.7 strains are grouped together in the same evolutionary branch, in the ML tree, we found that lineage 5 strains cluster with those in lineage 1.

### 3.2 Analysis of GP5 nucleotide homology

**Figure 3:**
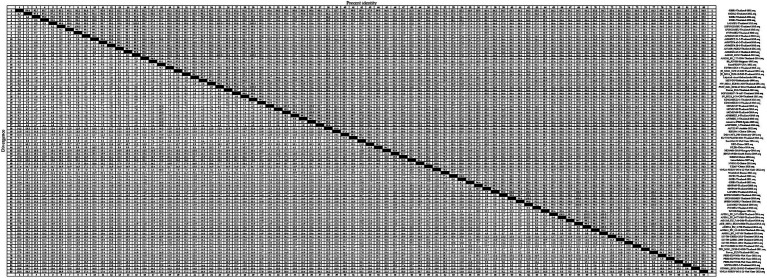
A total of 59 representative strains of each lineage of PRRSV-1 in Thailand and 24 classic representative PRRSV-1 strains from different countries were selected for PRRSV-1 GP5 nucleotide homology analysis.

**Figure 4:**
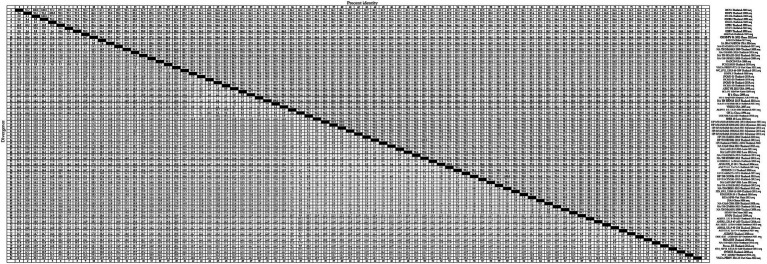
A total of 59 representative strains of each lineage of PRRSV-2 in Thailand and 24 classic representative PRRSV-2 strains from different countries were selected for PRRSV-2 GP5 nucleotide homology analysis.

To gain an insight into the genetic variation of the *GP5* gene in PRRSV since its introduction in Thailand, we selected 83 representative strains of PRRSV-1 (Figure 3) and 83 representative strains of PRRSV-2 (Figure 4) and used the respective GP5 sequences to perform nucleotide homology analysis.

For the PRRSV-1 GP5 sequences, we detected homologies ranging from 75.4 to 100.0% (Figure 3), among which, a clade D strain was found to show the lowest nucleotide homology. Nucleotide homology within the clade A strains ranged from 90.9 to 100.0%, among which, strains showing 100.0% nucleotide homology included: 1KPSN2S10EU-Thailand-2010 and 1VV0410EU-Thailand-2010; 1AS10EU-Thailand-2010 and AS1109_13EU-Thailand-2012; and 1AS10EU-Thailand-2010 and PCW_0611_EU12-27-D12-Thailand-2011, whereas AG04EU2.51-3-Thailand-2010 and EuroPRRSV-USA-2003 were identified as the two strains with the lowest homology of 90.9%. For clade D strains, nucleotide homologies ranged from 75.4 to 100.0%, among which, strains showing 100.0% nucleotide homology included 19CMU-07-Thailand-2019 and 19CMU-09-Thailand-2019; 19CMU-07-Thailand-2019 and Amervac PRRS-Spain-2009; and 19CMU-09-Thailand-2019 and Amervac PRRS-Spain-2009, whereas KZ2018-China-2018 and WestSib13-Russia-2013 were the strains with 75.4% nucleotide homology. For clade H strains, nucleotide homologies ranged from 84.7 to 100.0%, with 01CB1-Thailand-2001 and 01RB1-Thailand-2001; 1AF10EU-Thailand-2010 and 1PCN1H10EU-Thailand-2010; AF0311_EU_9-77-H05-Thailand-2014 and AS0311_EU_3-07-05-Thailand-2014 identified as those with 100.0% nucleotide homology, and PRRS-EU-VN11-Vietnam-2022 and SCP0311EU3-2-Thailand-2011 showing 84.7% homology.

For the PRRSV-2 GP5 sequences, we detected homologies ranging from 78.6 to 100.0% (Figure 4). VNUA-PRRSV-PT2-21-Vietnam-2021 and VNUA-PRRSV-TB1-23-Vietnam-2023 were found to show the lowest homology at 78.6%. Among these strains, those in lineage 1 showed nucleotide homologies ranging from 82.8 to 100.0%, with 00CS1-Thailand-2003 and 01UD6-Thailand-2003; 01NP1-Thailand-2003 and 02KK1-Thailand-2003; and 01UD6-Thailand-2003 and 02CB13-Thailand-2003 being among those showing 100.0% nucleotide homology, whereas CH-HNPY-01-2022-China-2022 and NA-TH-S001-2015-Thailand-2015 were found to show the homology at 82.8%. Lineage 5 strains showed nucleotide homologies ranging from 97.4 to 100.0%, among which, NA-TH-TRD018-2013-Thailand-2013 and NA-TH-TRT018-2013-Thailand-2013 were identified as strains with 100.0% homology, and 19CMU-03-Thailand-2019 and BJ-4-China-2000 were strains with 97.4% homology. Sublineage 8.7 strains showed nucleotide homologies ranging from 82.9 to 100.0%, among which, strains with 100.0% homology included HP-MYANMAR-0204AM1-2011-Myanmar-2011 and HP-MYANMAR-0204AM2-2011-Myanmar-2011; AF1211US_15-38-1-Thailand-2014 and AN1211US_15-41-4-Thailand-2014; and HP-TH-CCO034-2013-Thailand-2013 and NA-TH-CBI001-2013-Thailand-2013, and strains with 82.9% nucleotide homology were HP-TH-CCO034-2013-Thailand-2013 and VNUA-PRRSV-TB1-23-Vietnam-2023; and NA-TH-CBI001-2013-Thailand-2013 and VNUA-PRRSV-TB1-23-Vietnam-2023.

### 3.3 Analysis of GP5 amino acid homology

**Figure 5:**
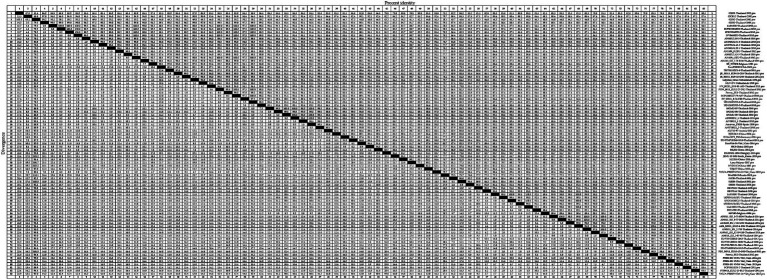
A total of 59 representative strains of each lineage of PRRSV-1 in Thailand and 24 classic representative PRRSV-1 strains from different countries were selected for PRRSV-1 GP5 amino acid homology analysis.

At the amino acid level, we detected homologies ranging from 41.3 to 100.0% for the GP5 sequences of PRRSV-1 strains (Figure 5). The strains 02SB2-Thaliand-2002 and Lena-Belarus-2007 have the lowest amino acid homology of 41.3%. Clade A strains showed amino acid homologies ranging from 41.6 to 100.0%, among which, strains with 100.0% homology included 1AS10EU-Thailand-2010 and 1KPSN2S10EU-Thailand-2010; 1PSN2S10EU-Thailand-2010 and AS1109_13EU-Thailand-2012; and PCW_0611_EU12-27-D12-Thailand-2011 and SCP1210EU7-79-A07-Thailand-2010, whereas 02BR1-Thailand-2002 and 02BR1-Thailand-2002 were identified as those strains with 41.6% amino acid homology. Among clade D strains, we detected amino acid homologies ranging from 76.7 to 100.0%. With 19CMU-07-Thailand-2019 and 19CMU-09-Thailand-2019; 19CMU-09-Thailand-2019 and AF0808EU_1-Thailand-2010; and 19CMU-07-Thailand-2019 and Amervac PRRS-Spain-2009 being among those strain with 100% homology, and HU19401-2016-Hungary-2016 and Lena-Belarus-2007 showing 76.7% homology. Clade H strain showed amino acid homologies ranging from 83.3 to 100.0%, among which strains with 100.0% amino acid homology included 1AF10EU-Thailand-2010 and AKE_S0311_EU10-6-B06-Thailand-2011; 2AG10EU-Thailand-2010 and Prevac_EU2-Thailand-2012; and AN0311_EU_2-708-Thailand-2014 and AS0311_EU_3-07-05-Thailand-2014, whereas 01RB1-Thailand-2001 and 3VC10EU-Thailand-2010 were identified as those strains with 83.3% homology.

**Figure 6:**
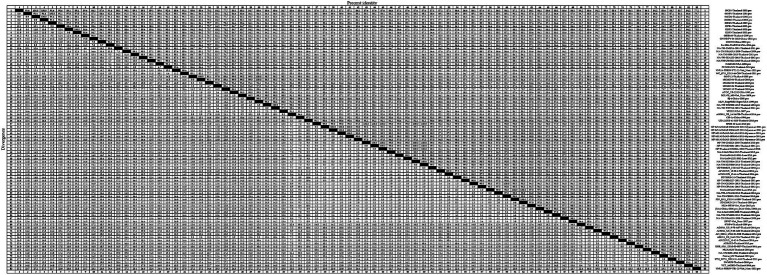
A total of 59 representative strains of each lineage of PRRSV-2 in Thailand and 24 classic representative PRRSV-2 strains from different countries were selected for PRRSV-2 GP5 amino acid homology analysis.

Among the GP5 sequences of the 83 assessed PRRSV-2 strains, we detected amino acid homologies ranging from 70.8 to 100.0% (Figure 6). The strains with amino acid homology of 70.8% were 19CMU-10-Thailand-2019 and VNUA-PRRSV-TB1-23-Vietnam-2023. For lineage 1 strains, homologies ranged from 84.7 to 100.0%, with 00CS1-Thailand-2003 and 01UD6-Thailand-2003; 01NP1-Thailand-2003 and 02KK1-Thailand-2003; and 02SP2-Thailand-2003 and 02SP3-Thailand-2003 being among those strains with 100.0% homology, with strains 02SP2-Thailand-2003 and CH-HNPY-01-2022-China-2022; CH-HNPY-01-2022-China-2022 and 02SP3-Thailand-2003 showing 84.7% homology. For lineage 5 strains, we detected amino acid homologies ranging from 88.6 to 100.0%, among which, 01NP1.2-Thailand-2005 and 19CMU-01-Thailand-2019; 19CMU-01-Thailand-2019 and MLV_RespPRRS-Repro-USA-1999; and NA-TH-TRD018-2013-Thailand-2013 and NA-TH-TRT018-2013-Thailand-2013 were found to have 100.0% homology, and 19CMU-10-Thailand-2019 and BJ-4-China-2000; 19CMU-10-Thailand-2019 and VR2332-USA-2007 were found to have a 88.6% homology. For sublineage 8.7 strains, amino acid homologies ranged from 77.2 to 100.0%. Strains with 100.0% homology included HP-TH-CCO034-2013-Thailand-2013 and NA-TH-CBI001-2013-Thailand-2013; NA-LAO-L167-2011-Laos-2011 and PIN_0311_US10-14-H09-Thailand-2011; and PIN_0311_US10-14-H09-Thailand-2011 and UD1210US-25-1-Thailand-2010, whereas UD1210US-61-E03-Thailand-2010 and VNUA-PRRSV-TB1-23-Vietnam-2023 showed a homology of 77.2%.

### 3.4 Amino acid sequence alignment

**Figure 7:**
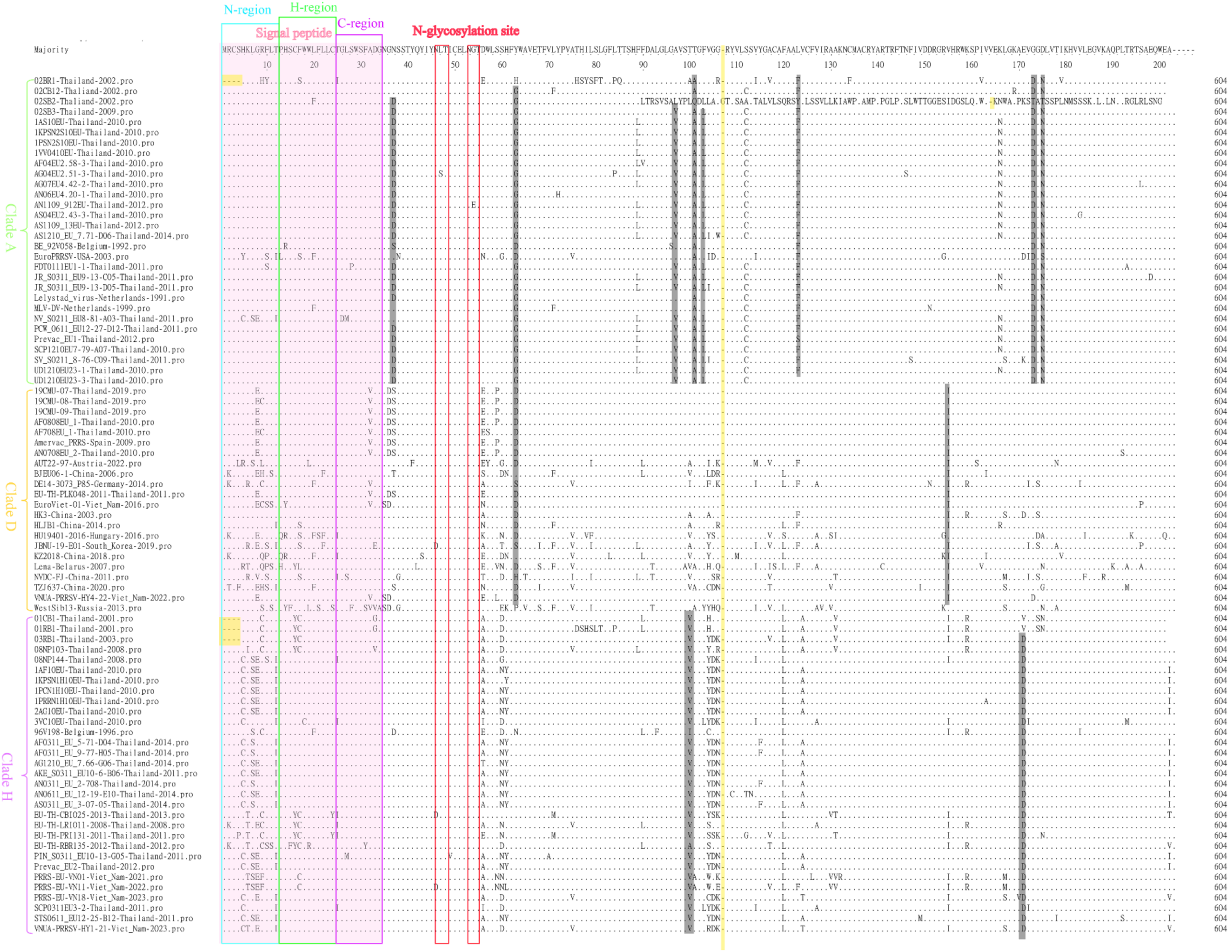
A total of 83 representative and prevalent strains in recent years were selected for PRRSV-1 GP5 amino acid sequence comparisons. GP5 amino acid sequences were analyzed using the sequence alignment editor MegAlign in DNASTAR software. A pink region represents the signal peptide; a yellow region represents an amino acid deletion or insertion; a gray region represents an amino acid mutation; a blue box represents an N-region, a green box represents an H-region, a purple box represents a C-region, and a red box represents an N-glycosylation site.

For comparisons of the GP5 amino acid sequences of the 166 selected PRRSV-1 and PRRSV-2 strains, we performed amino acid sequence alignment using the MegAlign sequence alignment editor in DNASTAR software, which accordingly revealed amino acid deletions, insertions, and mutations among the GP5 sequences of the PRRSV-1 strains (Figure 7). Among the PRRSV-1 isolates, we detected amino acid deletions at positions 1 to 4 in the GP5 of 02BR1-Thailand-2002, 01CB1-Thailand-2001, 01RB1-Thailand-2001, and 03RB1-Thailand-2003, whereas in strain 02SB2-Thailand-2002, we detected the insertion of glycine at position 107. In addition, multiple amino acid mutations were detected in PRRSV-1 GP5 sequences, with characteristic lineage-specific mutations occurring among clades A, D, and H strains. Notably, almost all assessed clade A strains were found to harbor the following mutations: N37 → D37, Y63 → G63, A97 → V97, T101 → A101, F103 → L103, L123 → F123, G173 → D173, and D175 → N175. Comparatively, in clade D strains, Y63 → D63 and V155 → I155 mutations tended to predominate, whereas T100 → V100 and A170 → D170 mutations were identified in all clade H strains. Notably, we detected no mutations at residues N46 and N53, which have been established to be glycosylation sites in PRRSV-1. N-glycosylation sites are generally well conserved and only a few strains have a single amino acid mutation at these sites. For example, EU-TH-CBI025-2013-Thailand-2013 and AN1109 912EU-Thailand-2012 have been found to have N46 → D46 and G54 → E54 mutations, respectively. Moreover, we detected widespread amino acid mutations in the N, H, and C regions of the signal peptide, although fewer overall mutations in these regions were detected among the clade A strains.

Amino acid sequence comparisons of the GP5 protein in PRRSV-2 strains revealed amino acid mutations at multiple sites, which were primarily detected in the following regions: B cell epitope, T cell epitope, primary neutralizing epitope (PNE), decoy epitope, and hypervariable region (HVR). Moreover, we detected lineage-specific mutations among the lineages 1, 5, and sublineage 8.7 strains. For example, virtually all strains in lineage 1 are characterized by L48 → I48 and A95 → I95, whereas all lineage 5 strains harbor S36 → D36, N37 → S37, Q59 → N59, and A95 → V95 mutations, and almost all sublineage 8.7/HP strain have A8 → C8, C25 → Y25, F26 → L26, L40 → I40, I162 → V162, and P201 → L201 mutations. Furthermore, all strains in sublineage 8.7/HP clade B harbor an E171 → G171 mutation, all strains in sublineage 8.7/HP JXA1 have an A30 → V30 mutation, and all sublineage 8.7/HP NA stains are characterized by F16 → S16, L40 → F40, and R152 → K152 mutations. Notably, in some strains, including 00CS1-Thailand-2003, 01UD6-Thailand-2003 and NA-TH-TRT018-2013-Thailand-2013, we detected the mutation of amino acid R13, a site that has been established to influence virulence. In addition, in the three strains CH-HNPY-01-2022-China-2022, NA-TH-CMI014-2011-Thailand-2011 and NADC30-USA-2008, we detected the mutation of amino acid N34, which has been identified as a glycosylation site in GP5 and tends to be conserved in most strains.

### 3.5 Recombination analysis

**Figure 8:**
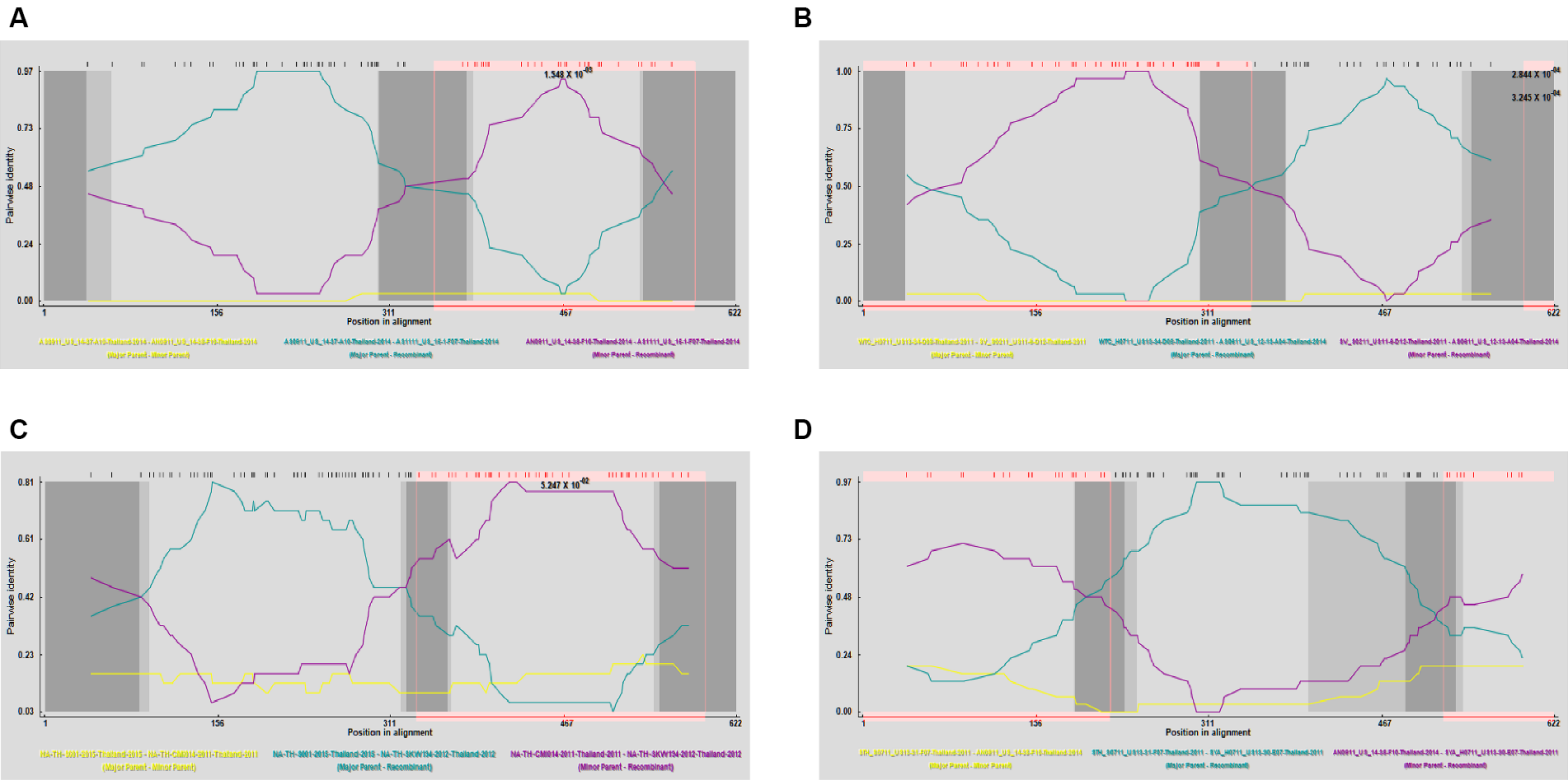
Detection of genetic recombination events in GP5 using recombination detection program (version 4.0). The *x*-axis represents a position in alignment, and the *y*-axis represents pairwise identity. Analysis results for recombinant strains AS1111_US_15-1-F07-Thailand-2014 **(A)**, AS0611_US_12-13-A04-Thailand-2014 **(B)**, NA-TH-SKW194-2012-Thailand-2012 **(C)**, and SYA_H0711_US13-30-E07-Thailand-2011 **(D)**.

To gain a more comprehensive understanding of GP5 recombination events that have occurred in PRRSV subsequent to its introduction in Thailand, we performed recombination analysis of the sequences of 726 *GP5* genes using RDP (version 4.0) software, which accordingly revealed three putative recombination events, which were supported by three or more algorithms with high confidence and statistical significance (*p* < 0.05) (Figure 8). Detailed information on these putative recombination events can be found in Table 1.

**Table 1:** Recombination analysis of the PRRSV *GP5* gene in Thailand.

Recombination event	Recombinant strains (lineages)	Major parental strain (lineages)	Minor parental strain (lineages)	Recombinant breakpoint	Recombination analysis method
1	AS1111_US_15-1-F07-Thailand-2014(8.7/NA)	AS0911_US_14-37-A10-Thailand-2014(8.7/NA)	AN0911_US_14-38-F10-Thailand-2014(8.7/HP)	301–387 (517–536)	RDP (*p* = 1.548 × 10^−03^) GENECONV (*p* = 1.941 × 10^−02^) BootScan (*p* = 1.211 × 10^−04^) MaxChi (*p* = 7.406 × 10^−06^) Chimera (*p* = 4.355 × 10^−06^) SiScan (*p* = 3.277 × 10^−06^) 3Seq (*p* = 1.712 × 10^−06^)
2	AS0611_US_12-13-A04-Thailand-2014(8.7/NA)	WTC_H0711_US13-34-D08-Thailand-2011(8.7/NA)	SV_S0211_US11-6-D12-Thailand-2011(8.7/HP)	303–381 (520–539)	MaxChi (*p* = 5.166 × 10^−05^) SiScan (*p* = 1.734 × 10^−12^) 3Seq (*p* = 1.705 × 10^−08^)
3	NA-TH-SKW194-2012-Thailand-2012(1)	NA-TH-S001-2015-Thailand-2015(1)	NA-TH-CMI014-2011-Thailand-2011(1)	320–366 (526–548)	BootScan (*p* = 1.633 × 10^−02^) MaxChi (*p* = 1.089 × 10^−04^) Chimera (*p* = 2.488 × 10^−05^) SiScan (*p* = 4.21 × 10^−04^) 3Seq (*p* = 2.331 × 10^−06^)
4	SYA_H0711_US13-30-E07-Thailand-2011(8.7/NA)	STH_S0711_US13-31-F07-Thailand-2011(8.7/NA)	AN0911_US_14-38-F10-Thailand-2014(8.7/HP)	401–540 (190–247)	MaxChi (*p* = 4.461 × 10^−04^) Chimera (*p* = 5.936 × 10^−04^) SiScan (*p* = 1.151 × 10^−04^) 3Seq (*p* = 3.924 × 10^−04^)

**Figure 9:**
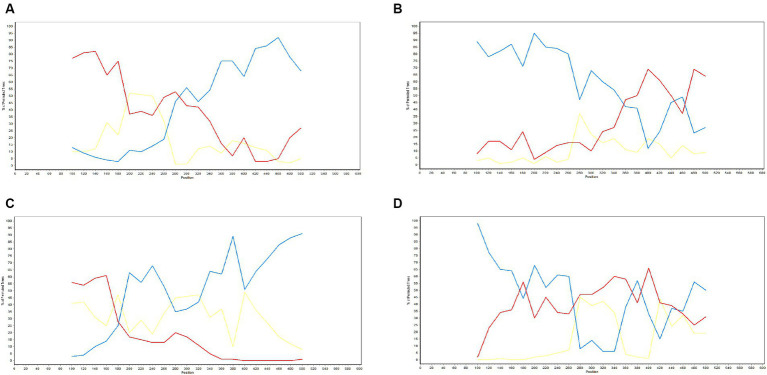
Validation of *GP5* gene recombination events using SimPlot (version 3.5.1). The *x*-axis represents position, and the *y*-axis represents the percentage of the tree arrangement. The red line represents the primary parent, the blue line represents the secondary parent, and the yellow line represents the reference strain. Validation results for the recombinant strains AS1111_US_15-1-F07-Thailand-2014 **(A)**, AS0611_US_12-13-A04-Thailand-2014 **(B)**, NA-TH-SKW194-2012-Thailand-2012 **(C)**, and SYA_H0711_US13-30-E07-Thailand-2011 **(D)**.

The potential recombination events were validated and confirmed using SimPlot (version 3.5.1), using which, we identified two recombination events (event 2 and event 4) (Figure 9). Event 2 was detected in recombinant strain AS0611_US_12-13-A04-Thailand-2014 (NCBI accession number: KJ954222.1), for which the major and minor parent strains are WTC_H0711_US13-34-D08-Thailand-2011 (NCBI accession number: JN848712.1) and SV_S0211_US11-6-D12-Thailand-2011 (NCBI accession number: JN848650.1), respectively. Recombination was found to have occurred in a region coinciding approximately with base pair sequences 303–381 and 520–539 within the *GP5* gene sequence. Event 4 was detected in the recombinant strain SYA_H0711_US13-30-E07-Thailand-2011 (NCBI accession number: JN848708.1), for which the major and minor parent strains were identified as STH_S0711_US13-31-F07-Thailand-2011 (NCBI accession number: JN848691.1) and AN0911_US_14-38-F10-Thailand-2014 (NCBI accession number: KJ954245.1), respectively. In this case, the region undergoing recombination occurred approximately at base pair sequences 190–247 and 401–540 within the *GP5* gene sequence.

## 4 Discussion

Since its emergence in Thailand, PRRSV has caused substantial damage to the country’s pig industry. Research conducted at the Chulalongkorn University Veterinary Diagnostic Laboratory has revealed that mixed infections of both PRRSV genotypes and continuous genomic variation of PRRSV can contribute immune escape, thereby making it difficult to control the disease using vaccines or stock management strategies (34). Furthermore, in addition to Thailand, the co-existence of genotype 1 and 2 strains has been identified in many parts of the world, and the lack of cross-protection against different strains has resulted in persistent infections (35). In this regard, amino acid variations in the highly variable regions (ORF5 and NSP2) of the virus genome have been a focal point of concern, and studies on the genetic variability of GP5 and its monitoring are of considerable importance.

As a consequence of genetic evolution, the PRRSV strains prevalent in Thailand have become increasingly diverse. Accordingly, continuous characterization and genetic analysis of PRRSV using a global systematic classification system are necessary to provide up-to-date genetic information for PRRS control and vaccine selection on an annual basis. In this study, a phylogenetic tree constructed using the NJ method based on the global PRRSV classification system revealed that both PRRSV-1 and PRRSV-2 are circulating in Thailand, with strains of the latter genotype being more prevalent, which is consistent with the findings reported by Nilubol et al. (14). Notably in this regard, however, earlier studies in 2004 reported a predominance of PRRSV-1 isolates in Thailand, which were more commonly observed than PRRSV-2 strains (10, 36, 37), whereas in their 2015 study, Chaikhumwang et al. (38) detected a reversal in the pattern of dominance, with genotype 2 strains gaining a predominant status. This transition was subsequently corroborated by Jantafong et al. (39), who analyzed the genetic diversity of Thai PRRSV isolates from 2008 to 2013. The NJ phylogenetic tree constructed in the present study also revealed that the PRRSV-1 strains currently circulating in Thailand can be divided into three evolutionary branches, namely, clades A, D, and H. Notably, the PRRSV-1 reference strains used in this study, which were mainly clade D strains selected from other countries, are genetically relatively distant from the PRRSV-1 strains circulating in Thailand, which would tend to indicate that these strains have undergone significant variation subsequent to their introduction into Thailand. Moreover, we found that clade H Thai strains are characterized by a close genetic relationship with selected reference strains from Vietnam, which we speculate could be ascribed to the geographical proximity of the two countries, which would presumably facilitate the inter-country transmission of PRRSV.

In the global PRRSV classification system, PRRSV-2 strains are divided into nine lineages based on ORF5 sequences (27, 40, 41). The PRRSV-2 strains circulating in Thailand are classified as lineages 1, 5, and sublineage 8.7 strains, with sublineage 8.7 comprising sublineages 8.7/HP and 8.7/NA strains, the former of which can be further sub-divided into clade A (SX2009-like), clade B (09HEN1-like), and JXA1-like isolates. The lineage assignment of PRRSV-2 in Thailand tends to be indicative of different modes of introduction of PRRSV into the country. Among the PRRSV-2 strains circulating in Thailand, those of sublineage 8.7/NA are currently predominant, and those lineage 5 are genetically close to strain of sublineages 8.7 and 8.7/HP. This contrasts with the profile of PRRSV-2 strains introduced early in Thailand in 2003, a majority of which were of a lineage 1 type, which tend to be closely related to selected reference strains from the United States, China, and Vietnam, thus providing evidence of the spread of PRRSV among different countries. However, Nilubol et al. (42) have found that the development of PRRSV isolates in Thailand is not influenced by geography. Strains such as 01NP1 and 02KK1, 00CS1 and 01UD6, and 01CB1 and 01RB1 within lineage 1 were collected from different provinces in Thailand, and phylogenetic tree analysis in our study reveals that these strains are genetically close and do not show significant geographical differences. In this regard, it is also worth noting that sublineage 8.7/HP clade A strains show a close genetic relationship with selected reference strains from Myanmar and Laos, thus indicating that either the PRRSV strains detected in Thailand may have been introduced from these two countries or that there have been interchanges of strains among countries within the entire Southeast Asian region. Clade JXA1-like strains include JXA1-China-2006, SRV07-Vietnam-2007, and NA-CAM-C008-2008-Thailand-2008, which provide evidence to indicate the spread and prevalence of PRRSV in terms of year, country, and geographic location. In addition, we established that the selected reference strains from the past 5 years exhibit a close genetic relationship with Thai PRRSV strains, thereby placing the variation and recombination of PRRSV in Thailand in a global context.

Although the ML phylogenetic tree constructed in this study tended to be consistent with that obtained using the NJ method, we did note certain differences. For example, compared with the NJ tree, we observed greater the genetic distances between the reference strains from Myanmar and clade A and JXA1-like strains in the ML tree. In addition, whereas in the NJ tree, lineage 5 and sublineage 8.7 are grouped on the same evolutionary branch, in the ML tree, lineage 5 strains are clustered on the same evolutionary branch as lineage 1 strains. We speculate that these discrepancies could be attributable to differences in the algorithms used and model assumptions.

Analyses of the nucleotide and amino acid homologies of PRRSV-1 GP5 sequences revealed homologies ranging from 75.4 to 100.0% and from 41.3 to 100.0%, respectively. In terms of clade distribution, we obtained values of between 90.9 and 100.0%, 75.4 and 100.0%, and 84.7 and 100.0% for the nucleotide homologies among the clades A, D, and H strains, respectively, thereby revealing a comparatively higher degree of homology among the clade A strains. With respect to amino acid homologies, we obtained values of between 41.6 and 100.0%, 76.7 and 100.0%, and 83.3 and 100.0% for clades A, D, and H strains, respectively. These values accordingly reveal lower homologies of PRRSV-1 GP5 sequences at the amino acid level compared with the nucleotide level, particularly for that between the strains 02SB2-Thaliand-2002 and Lena-Belarus-2007, which have an amino acid homology of only 41.3%. These findings accordingly tend to indicate a lower likelihood of PRRSV-1 strains in Thailand being introduced from Belarus. Conversely, our findings that strains 19CMU-07-Thailand-2019 and Amervac PRRS-Spain-2009 within clade D exhibit 100.0% nucleotide homology, and that strains 19CMU-09-Thailand-2019 and Amervac PRRS-Spain-2009 exhibit 100.0% nucleotide and amino acid homologies would tend to indicate a higher likelihood that Thai PRRSV-1 strains have been introduced from Spain.

The nucleotide and amino acid homology analyses performed for the PRRSV-2 GP5 sequences revealed homologies ranging from 78.6 to 100.0% and 70.8 to 100.0%, respectively. In terms of the individual PRRSV-2 lineages, we obtained nucleotide homology values of between 82.8 and 100.0%, 97.4 and 100.0%, and 82.9 and 100.0% for the strains of lineages 1, 5, and sublineage 8.7, respectively. Among these strains, we obtained a value of 78.6% for the nucleotide homology between the strains VNUA-PRRSV-PT2-21-Vietnam-2021 and VNUA-PRRSV-TB1-23-Vietnam-2023, thereby is indicating significant variation in PRRSV-2 strains in Vietnam between 2021 and 2023. Among PRRSV-2 strains, a value of 100% was obtained for the nucleotide homology between GXN32-China-2023 and NA-TH-TRT018-2013-Thailand-2013, which indicates a high likelihood that PRRSV-2 strains in Thailand have been introduced to China. Within sublineage 8.7, strains showing 82.9% nucleotide homology include HP-TH-CCO034-2013-Thailand-2013 and VNUA-PRRSV-TB1-23-Vietnam-2023; and NA-TH-CBI001-2013-Thailand-2013 and VNUA-PRRSV-TB1-23-Vietnam-2023, which provides evidence to indicate that strains in Vietnam newly detected in 2023 may have evolved from PRRSV strains in Thailand. With respect to amino acid homologies among the PRRSV-2 GP5 sequences, we obtained values of between 84.7 and 100.0%, 88.6 and 100.0%, and 77.2 and100.0% for lineage 1, 5, and sublineage 8.7 strains, respectively. Among the strains, 08RB160-Thailand-2009 and 19CMU-10-Thailand-2019 were identified with 74.8% amino acid homology, which thus indicates a significant variation among strains in Thailand between 2009 and 2019 at the amino acid level. Within PRRSV-2, we detected a 100% amino acid homology between 19CMU-01-Thailand-2019 and MLV_RespPRRS-Repro-USA-1999, the latter strain of which is a modified live vaccine (MLV) of a USA genotype, thereby revealing the presence of vaccine-derived viruses among the lineage 5 strains detected in Thailand (43). Conversely, we detected only a 70.8%, amino acid homology between the strains 19CMU-10-Thailand-2019 and VNUA-PRRSV-TB1-23-Vietnam-2023, which does not support the conclusion that strains newly discovered strains in Vietnam in 2023 may have evolved from strains in Thailand. Within sublineage 8.7, the strains with 100.0% amino acid homology include NA-LAO-L167-2011-Laos-2011 and PIN_0311_US10-14-H09-Thailand-2011, which may provide evidence for the bidirectional transmission of sublineage 8.7 strains between Thailand and Laos in 2011. UD1210US-61-E03-Thailand-2010 and VNUA-PRRSV-TB1-23-Vietnam-2023 were identified as those sublineage 8.7 strains with the lowest amino acid homology (77.2%), once again indicating a relatively low homology between the newly discovered strains in Vietnam in 2023 and Thai strains at the amino acid level.

The GP5 protein, which has been established to play important roles during the assembly and replication of PRRSV virus particles in susceptible cells (32, 44, 45), comprises essential immunogenic domains and peptide or protein motifs associated with virus-mediated neutralization, including signal peptides, transmembrane regions, antigenic clusters, and glycosylation sites, which contribute to determining the biological functions of PRRSV (46). Amino acid deletions, insertions, and mutations in the PRRSV GP5 sequence can have a pronounced influence on viral virulence and proliferation, as well as the epidemiology and spread of PRRS, and indeed, the 2006 outbreak of HP-PRRSV in China was found to coincide with a discontinuous deletion of 30 amino acids in NSP2, although subsequent studies confirmed that this NSP2 deletion showed no direct correlation with viral virulence (7, 47, 48). However, Chen et al. (49) have demonstrated that unique deletions and insertions in GP5 have resulted in the emergence of novel PRRSV variants with distinctive genetic features and high pathogenicity, whereas from their phylogenetic analysis based on the amino acid sequence of GP5, Sun et al. (50) revealed that a novel NADC30-like PRRSV with a unique single amino acid deletion at the 34 position had widely circulated and evolved into a new sublineage. Furthermore, Chaikhumwang et al. (38) have suggested that the evolution of Thai PRRSV-1 viruses from cluster II to cluster III might be associated with immune evasion induced by amino acid substitutions at N-linked glycosylation sites. In the present study, we identified amino acid deletions, insertions, and mutations in sequences of PRRSV-1 GP5, with strains 02BR1-Thailand-2002, 01CB1-Thailand-2001, 01RB1-Thailand-2001, and 03RB1-Thailand-2003 being characterized by amino acid deletions at positions 1 to 4. These strains were all discovered in Thailand during the early years following the initial detection of PRRSV. As the spread and prevalence of Thai strains are not influenced by geographical location, some Thai strains from different geographical locations during this particular period share this deletion feature. Furthermore, in addition to amino acid mutations, we found that strain 02SB2-Thailand-2002 is characterized by a glycine insertion at position 107, and we speculate that this insertion event and the associated mutations may have influenced the genetic variation of subsequent Thai strains. The analysis also found widespread occurrence of characteristic mutations within lineages, which may underlie lineage classification and indirectly suggest differences among the ancestral strains of different lineages. In addition, we identified the conserved nature of the N46 and N53 amino acids at PRRSV-1N-glycosylation site, at which no amino acid mutations had occurred. In Figure 7, the red box highlights the general conservation of N-glycosylation sites, with only a few strains exhibiting single amino acid mutations. Contrastingly, The N, H, and C regions of the signal peptide were found to be characterized by widespread amino acid mutations, although clade A strains tended to have fewer mutations in these regions (Figure 7).

**Figure 10:**
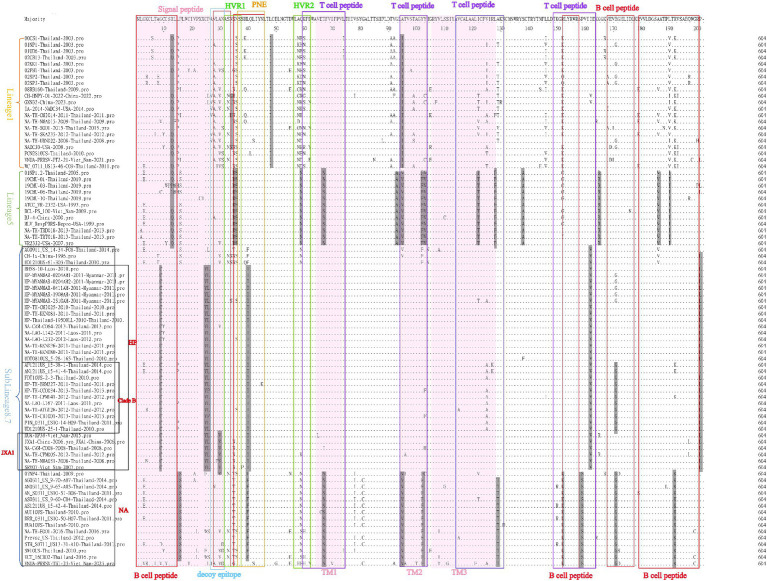
A total of 83 representative and prevalent strains in recent years were selected for PRRSV-2 GP5 amino acid sequence comparisons. GP5 amino acid sequences were analyzed using the sequence alignment editor MegAlign in DNASTAR software. A pink area represents a signal peptide; a gray area represents amino acid mutations; a red box represents a B cell peptide; a blue box represents a decoy epitope; a green box represents a hypervariable region; a yellow box represents a primary neutralizing epitope; and a purple box represents a T cell peptide.

With respect to the amino acid sequences of PRRSV-2 GP5, we detected mutations in B cell epitope, T cell epitope, PNE, decoy epitope, and HVR regions (Figure 10). Zhou et al. (51) demonstrated that the *GP5* gene of the SCcd17 strain encodes a 199 amino acid-protein with a novel single amino acid deletion at position 33 in the HVR1 region, which was not found in this study. Additionally, similar to PRRSV-1 strains, we detected lineage-specific amino acid mutations among the lineages 1, 5, and sublineage 8.7 strains of PRRSV-2. Notably, the five reference strains from Myanmar had several identical amino acid mutations in common with sublineage 8.7/HP strains, thereby indicating a degree of similarity and potential interchange between strains from Thailand and Myanmar. Furthermore, in a few strains, including 00CS1-Thailand-2003, 01UD6-Thailand-2003, and NA-TH-TRT018-2013-Thailand-2013, we detected a mutation at amino acid position R13, which has been established to influence virulence. In addition, whereas in a majority of strains, the glycosylation site N34 in GP5 tends to be highly conserved, we detected three strains (CH-HNPY-01-2022-China-2022, NA-TH-CMI014-2011-Thailand-2011, and NADC30-USA-2008) with mutations at this site. Overall, the GP5 protein is characterized by a high degree of variability, and these observed mutations are assumed to provide a driving force for the continual evolution of PRRSV.

Another further important phenomenon contributing to the evolution of PRRSV is genetic recombination, as highlighted by the emergence of HP-PRRSV in China (11, 52, 53). Furthermore, the findings of several studies have indicated that PRRSV strains can generate novel lineages or sub-lineages via different types of recombination event between members (54–57). Since 1996, PRRSV has been continuously spreading throughout Thailand, during which time, strains have undergone frequent amino acid mutations and recombination events. In this study, we conducted recombination analysis for the *GP5* gene of 726 PRRSV strains, and thereby identified three potential recombination events, the veracity of which was supported by three or more algorithms with statistical significance (*p* < 0.05). Two of these events (event 2 and event 4) were confirmed based verification using SimPlot (version 3.5.1). Strain AS0611_US_12-13-A04-Thailand-2014 (NCBI accession number: KJ954222.1) was identified as an Event 2 recombinant, for which the major and minor parental strains were identified as WTC_H0711_US13-34-D08-Thailand-2011 (NCBI accession number: JN848712.1) and SV_S0211_US11-6-D12-Thailand-2011 (NCBI accession number: JN848650.1), respectively. The recombination event was estimated to have occurred in the vicinity of the 303–381 and 520–539 bp sequences of the *GP5* gene. Similarly, SYA_H0711_US13-30-E07-Thailand-2011 (NCBI accession number: JN848708.1) was identified as the recombinant strain derived from the event 4 recombination, for which the assumed the major and minor parent strain were STH_S0711_US13-31-F07-Thailand-2011 (NCBI accession number: JN848691.1) and AN0911_US_14-38-F10-Thailand-2014 (NCBI accession number: KJ954245.1), respectively. In this case, the region undergoing recombination was estimated to have occurred in the vicinity of the 190–247 and 401–540 bp sequences of the *ORF5* gene. In both cases, these events involved recombination between strains grouped on the NA and HP branches within sublineage 8.7. Accordingly, given that of the two PRRSV genotypes, PRRSV-2 is more prevalent in Thailand, and sublineage 8.7 is the major circulating lineage of PRRSV-2, the findings of our recombination analysis indicate that greater attention should perhaps be paid to the genetic evolution of PRRSV epidemic lineages in Thailand.

Given that the GP5 protein has been established to play important role in influencing the invasion, replication, and pathogenicity of PRRSV, and thereby the transmission and prevalence of the virus, this protein could also as a promising target for the development of drugs and diagnostic reagents. In this regard, Wu et al. (58) have demonstrated that GP5 glycoprotein modified with Fc tags is an effective inducer of PRRSV-specific neutralizing antibodies, thereby highlighting the promising potential utility of GP5 in vaccine development. In addition to such development, it will be essential maintain continuous monitoring of the highly variable GP5 protein in order to constant update our understanding of the perpetually evolving epidemiological dynamics of PRRSV in Thailand, thereby making a valuable contribution to the prevention and control of future PRRS outbreaks.

## 5 Conclusion

Both PRRSV-1 and PRRSV-2 are prevalent in Thailand, with PRRSV-2 strains being characterized by a wider distribution. In the phylogenetic tree constructed using the NJ method, PRRSV-1 strains circulating in Thailand are divided into three evolutionary branches, namely, clades A, D, and H, whereas the prevalent PRRSV-2 strains can be broadly classified into the three groups, including lineages 1, 5, and sublineage 8.7. Sublineage 8.7 strains include those in sublineage 8.7/HP and sublineage 8.7/NA, the former of which can be further sub-divided into clade A, clade B, and JXA1-like strains. Among these PRRSV-2 strains, those in sublineage 8.7/NA are currently the most predominant strains circulating in Thailand, and lineage 5 strains were found to be genetically closer to those in sublineages 8.7 and 8.7/HP. Phylogenetic trees constructed using the ML method revealed evolutionary pattern that were generally consistent with those obtained using the NJ method. Homology analysis revealed nucleotide and amino acid homologies among the *GP5* gene of PRRSV-1 of strains ranging from 75.4 to 100.0% and 41.3 to 100.0%, respectively, and those among the *GP5* gene of PRRSV-2 GP5 strains ranging from 78.6 to 100.0% and 70.8 to 100.0%, respectively. The estimates evolutionary distances with respect to reference strains obtained from the phylogenetic tree and homology analysis provide insights into the transmission trends of PRRSV strain detected in Thailand and other countries. On the basis of amino acid sequence alignment, we identified amino acid mutations, insertions, and deletions in the sequences of PRRSV-1 GP5, and amino acid mutations in PRRSV-2 GP5 sequences, with some of the mutated sites being identified as key amino acid positions involved in the biological functions of GP5. Furthermore, recombinant analysis of the 726 strain sequences revealed two recombination events that have occurred within the PRRSV-2 sublineage 8.7 strains. The findings of our mutation and recombinant analyses accordingly confirm the high variability of the GP5 protein in PRRSV.

## Data availability statement

The datasets presented in this study can be found in online repositories. The names of the repository/repositories and accession number(s) can be found in the article/Supplementary material.

## Author contributions

YZ: Conceptualization, Formal analysis, Methodology, Writing – original draft. GL: Conceptualization, Data curation, Formal analysis, Writing – original draft. KL: Conceptualization, Formal analysis, Methodology, Writing – original draft. QL: Conceptualization, Data curation, Methodology, Writing – original draft. WS: Project administration, Supervision, Writing – review & editing. MZ: Funding acquisition, Investigation, Project administration, Supervision, Writing – review & editing.

## Footnotes

^1^https://itol.embl.de
